# Supplementary material for: Microcantilever-integrated photonic circuits for broadband laser beam scanning
Source: Nat Commun. 2023 May 8;14:2641. doi: 10.1038/s41467-023-38260-8 (PMC10167362; doi:10.1038/s41467-023-38260-8)
Supplement: Supplementary file 1 — Supplementary information [file 41467_2023_38260_MOESM1_ESM.pdf]

Microcantilever-integrated photonic circuits for broadband laser beam scanning: Supplementary information

Saeed Sharif Azadeh,[1] Jason C. C. Mak,[2] Hong Chen,[1] Xianshu Luo,[3] Fu-Der Chen,[1,2] Hongyao Chua,[3] Frank Weiss,[1] Christopher Alexiev,[2] Andrei Stalmashonak,[1] Youngho Jung,[1] John N. Straguzzi,[1] Guo-Qiang Lo,[3] Wesley D. Sacher,[1] & Joyce K. S. Poon[1,2]

[1] Max Planck Institute of Microstructure Physics, Weinberg 2, 06120 Halle, Germany

[2] University of Toronto, Department of Electrical and Computer Engineering, 10 King's College Road, Ontario, Toronto, Canada

[3] Advanced Micro Foundry Pte. Ltd., 11 Science Park Road, Singapore Science Park II, 117685, Singapore

1. Simulation and characterization of the grating couplers

The same 25 µm long grating coupler design was used in all devices presented in the manuscript. The grating coupler was fabricated by fully etching the 150 nm thick SiN waveguide layer. The width of the grating coupler was 10 µm. A 200 µm long adiabatic taper with an initial width of 380 nm (waveguide width) and a final width of 10 µm (grating coupler width) ensured only the fundamental TE mode was launched into the grating coupler. The lengths of the teeth and grooves were 220 nm. Figure S1a shows the simulated far-field pattern of the grating output beam at $\lambda = 488$ nm. The simulations were performed using three-dimensional FDTD in Lumerical. The far-field pattern along the longitudinal axis (cutline A in Fig. S1a) is plotted in Fig. S1b, and shows a full-width-half-maximum (FWHM) of 0.78° for the output beam. Similarly, the far-field pattern along the transverse direction (cutline B in Fig. S1a) predicts an FWHM of 2.2° as shown in Fig. S1c. As mentioned in the manuscript, the measured far-field image of the grating coupler had an FWHM of 1.4° and 3.1°, respectively in longitudinal and transverse directions. The difference between the simulation and experimental results can be attributed to fabrication variation, as the teeth width of 220 nm was close to the minimum feature size.

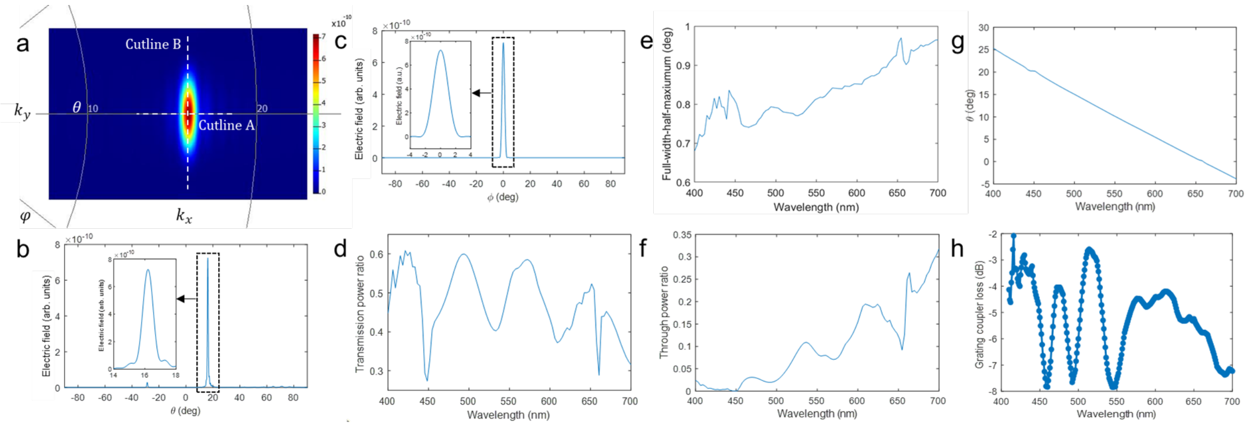

Fig. S1: Grating couplers performance. **a** Simulated far-field pattern of the grating coupler output at λ=488 nm. **b** one-dimensional representation of the output beam along Cutline A (k_x). **c** One-dimensional representation of the output beam along Cutline B (k_y). **d** Simulated off-chip transmitted power of the grating coupler normalized to input power. **e** Simulated full-width-half-maximum (FWHM) of the grating coupler output. **f** Simulated on-chip optical power remaining at the end of the grating coupler normalized to the input power. **g** Simulated output beam angle of the grating coupler. **h** Measured optical loss of the grating couplers.

Figure S1d shows the simulated optical power transmission of the grating coupler normalized to the launch power in the wavelength range between 400 and 700 nm. Peaks and valleys in the transmission spectrum of the grating are expected due to the interference between the emitted beam and the back-reflected beam from the substrate. Based on our simulations, the depth of the fluctuations can be minimized by reducing the distance between the grating coupler and the substrate. The simulated FWHM spectrum of the output beam along the propagation direction is shown in Fig. S1e, and is $< 1°$ in the visible spectrum. The ratio of the power remaining at the end of the 25 μm long grating coupler is negligible at $\lambda = 488$ nm, as shown in Fig. S1f. The simulated wavelength dependence of the output beam angle is shown Fig. S1g, which is in good agreement with the measured value of $\sim 0.1°$/nm. Finally, the measured transmission spectrum of the grating coupler is shown in Fig. S1h, which is slightly different from the simulated transmission spectrum (Fig. S1d) in terms of optical loss at a given wavelength. The discrepancy can be due to the high sensitivity of the optical transmission spectrum to the thickness of the oxide. For this measurement, we used a supercontinuum laser source coupled to an external tunable optical filter with a 1 nm optical bandwidth. At each wavelength, the polarization of the launch beam is set to TE to maximize the transmission of the grating coupler.

2. Selection of layer thicknesses for the Al-SiO_2 bimorph cantilever

Generally, bending can be induced in a two-material laminate by a difference in their thermal expansion coefficients. Bimaterial (or bimorph) cantilevers have been extensively studied in the context of bimetallic

thermostats, such as the classic result of Timoshenko [1], and more recently as MEMS actuators that use metal and Si [2] or SiO_2 [3–6]. Bimaterial cantilevers can be formed in silicon (Si) photonic platforms by repurposing existing process features. Si photonic platforms typically include a thick top Al layer for electrical routing and bond pads that is on top of the SiO_2 cladding. As well, undercut etches remove a portion of the substrate to suspend the oxide and top metal layers. Undercut trenches are typically used for improving thermal isolation for thermo-optic phase shifters [7] and suspended edge couplers [8]. Bimorph cantilevers can be formed by an undercut under a region with a top metal layer. Optical circuits can be incorporated inside the cantilevers.

The deflection, *d*, of an Al-SiO_2 bimorph cantilever with the same base and tip width follows the equation [9]:

$$d = \frac{cx(1+x)^2}{c^2x^4+4cx^3+6cx^2+4cx+1}\frac{3\Delta\alpha\Delta T L^2}{t_{Al}+t_{SiO2}},$$

where $x = \frac{t_{Al}}{t_{SiO2}}$ is the ratio of the thicknesses of Al and SiO_2, $c = \frac{E_{Al}}{E_{SiO2}}$ is the ratio of the Young's modulus, $\Delta\alpha = \alpha_{Al} - \alpha_{SiO2}$ is the difference of thermal expansion coefficients, ΔT is the temperature change from initial, and L is the cantilever length. The deflection is maximized when the thickness ratio of the metal to elastic material is of the ratio [9]

$$\frac{t_{Al}}{t_{SiO2}} = \sqrt{\frac{E_{SiO2}}{E_{Al}}}.$$

For values assumed in our numerical simulations, $E_{Al} = 70\ GPa$, $E_{SiO2} = 73\ GPa$, $\frac{t_{Al}}{t_{SiO2}} = 1.02$. In our platform, due to compromise with other features, this ratio was $\frac{t_{Al}}{t_{SiO2}} = 0.8$. Based on the deflection equation, this nonoptimal ratio is expected to decrease the maximum deflection by 1.5%. The deflection in the measured device will also be limited by the yield strength at the highest temperature reached. Alloys of Al which are CMOS compatible can be investigated to further improve reliability and range of the deflection. Beyond cantilevers, advanced bimorph actuator geometries and structures such as those in [6] can also be combined with integrated photonics in the future.

3. Thermal and deflection simulations of the cantilevers

3.1 Rectilinear cantilevers

Figure S2a shows the simulated temperature along the 500 µm long rectilinear cantilever under 10 mW of applied electrical power. The average temperature of the cantilever for a given electrical power is inversely proportional to the cantilever volume. Hence, reducing the thickness and width of the device results in a

higher power efficiency. However, a narrower width worsens the mechanical robustness of the cantilever, especially for longer devices. Moreover, the thickness of the SiO_2 must be determined to suit the functionalities of other devices on the platform such as bilayer directional couplers. Shorter rectilinear cantilevers have a lower power efficiency due to the heat-sinking provided by the metal lines that form the electrical connections on the clamped side of the cantilever. Fig. S2b shows the simulated temperature along the 500 µm long cantilever is lower near the clamped end. Fig. S2c plots the displacement along the cantilever, and for an applied electrical power > 15 mW, its distal end comes into contact with the substrate, and the cantilever starts to bow upward near its center. This effect leads to the change in the slope of the angular tuning curves in Fig. 3c of the main manuscript.

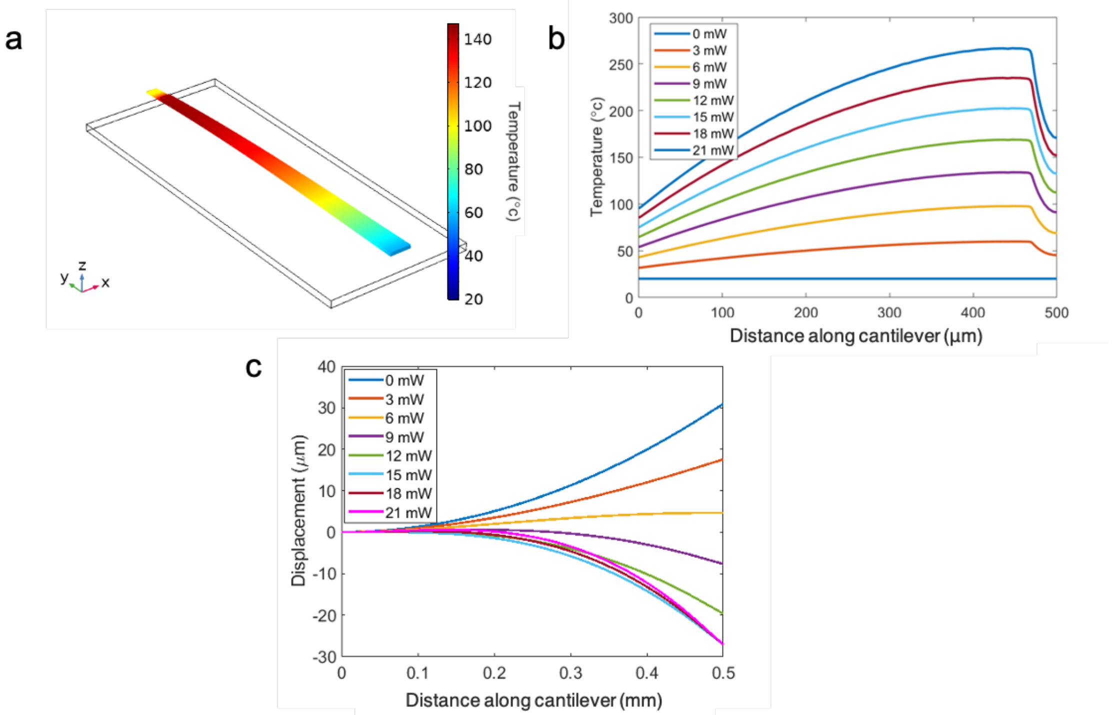

Fig. S2: Electro-thermal simulation of a 500 µm long rectilinear cantilever. a Simulated temperature distribution under applied electrical power of 10 mW. **b** Calculated temperature and **c** displacement along the cantilever for different electrical powers.

3.2 L-shaped cantilevers

In an ideal 2D beam steering system, the steering of the output beam in the longitudinal direction (θ) should be fully independent of the control voltage of the transverse direction (V_φ), and vice versa. For the L-shaped cantilever, this means that the electrical power applied to the secondary arm (P_θ) should not tilt the grating coupler in the transverse direction. However, as shown in Fig. S3a, the temperature of the secondary arm under 10 mW electrical power has a tail that extends to the primary arm on the left side, causing it to slightly bend down and resulting in a parasitic angular tilt ($\Delta\varphi$) in the transverse direction. The resulting $\Delta\varphi$ is

calculated and shown in Fig. S3b. Nevertheless, the angular tilt in the desired direction (as shown in Fig. 4b, c) dominates the tilt due to the thermal crosstalk between the arms, and thus the beam can still scan over a wide range (Fig. 4d) in Fourier space. As shown in Fig. S3a, the thermal crosstalk between the secondary arm and the left primary arm is higher compared to the primary arm on the right side, due to the metal connection between these two arms electrically connect the secondary arm. Similarly, applying electrical power to the primary arms results in a slight temperature increase in the secondary arm (Fig. S3c) and the corresponding angular tilt in the longitudinal direction (Fig. S3d).

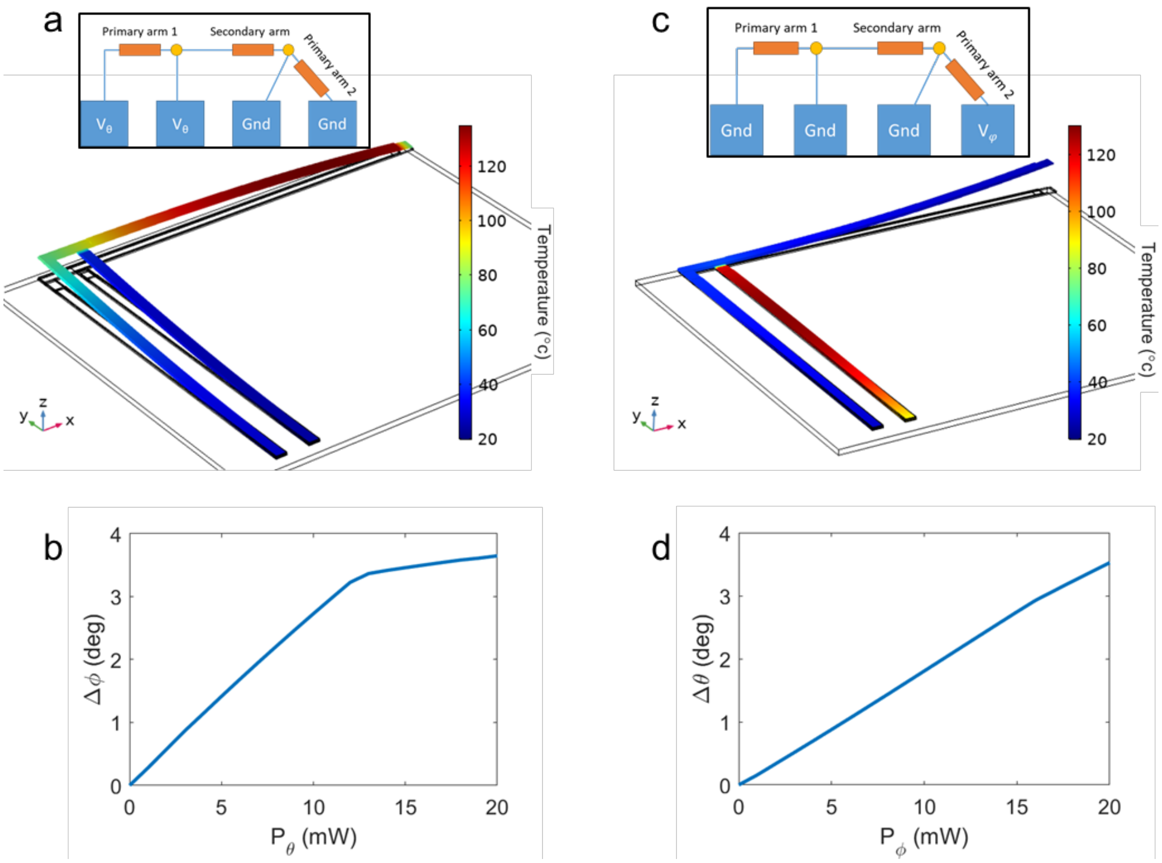

Fig. S3: Electro-thermo-mechanical simulation of the L-shaped cantilever. a Simulated temperature overlaid on the displaced L-shaped cantilever when electrical power of P_θ = 10 mW is applied to the secondary arm. **b** Simulated angular tilt of the grating coupler in transverse direction under the electrical power applied to the secondary arm. **c** Simulated temperature of the L-shaped cantilever overlaid on its displacement under electrical power of P_φ = 10 mW applied to the right-hand side primary arm. **d** Calculated θ-axis angular tilt of the grating coupler as a result of the voltage applied to the right primary arm. The insets in **a** and **c** show the circuit diagrams.

4. Time response measurements

Using the procedure described in the Methods Section of the main manuscript, we measured the temporal response of several cantilevers. Fig. S4a shows a few recorded far-field trajectories of the 800 μm long

rectilinear cantilevers, taken with different duty cycles. In the measurements in Fig. S4a, the period of the signal was set to 20 ms. The measured and simulated time responses of the shortest rectilinear cantilever are shown in the main manuscript (Fig. 3b). Figures S4b, c, d show the extracted rise time and fall times of the 500 μm, 800 μm, and 1 mm long rectilinear cantilevers. In these measurements, the applied square pulse had a period of 20 ms and the duty cycle was varied between 1% to 50% to measure the rise time, and 50% to 99% to measure the fall time. Since the temporal responses of the devices were measured at discrete points (blue dots in Fig. S4), we fit an exponential curve to the measured values (red dashed lines). The rise time (/fall time) of the cantilevers were measured to be 1.01 (/1.41), 2.42 (/2.84), 4.14 (/4.03), and 3.63 (/5.72) ms respectively for 300, 500, 800, and 1000 μm long cantilevers, in good agreement with the simulated (yellow dashed lines in Fig. S4) time response.

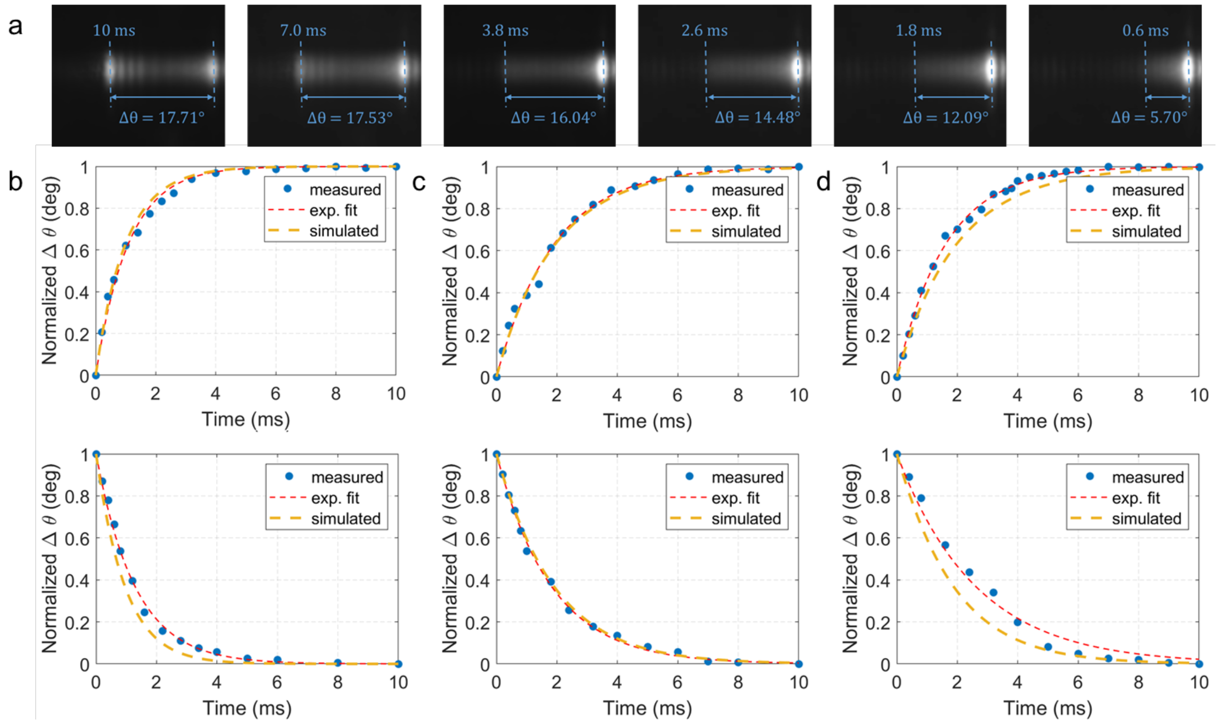

Fig. S4: Time response of the rectilinear cantilevers. a Recorded far-field images of the output of the 800 μm long rectilinear cantilever, under applied square signal with maximum power of 10 mW, period of 20 ms, and duty cycles of 50%, 35%, 19%, 13%, 9% and 3%, respectively, from left to right. **b, c, d** Measured and simulated rise time (top) and fall time (bottom) of the 500 μm (**b**), 800 μm (**c**), and 1 mm (**d**) long cantilevers.

We also measured the time responses of the L-shaped cantilevers. The results are shown in Figs. S5. In this case, we separately measured the temporal response of the secondary arm (Fig. 5Sa) and the right-hand side primary arm (Fig. S5b). We measure the rise time of 4.26 and 4.65 ms and fall time of 4.87 and 5.64 ms respectively for the primary and the secondary arms.

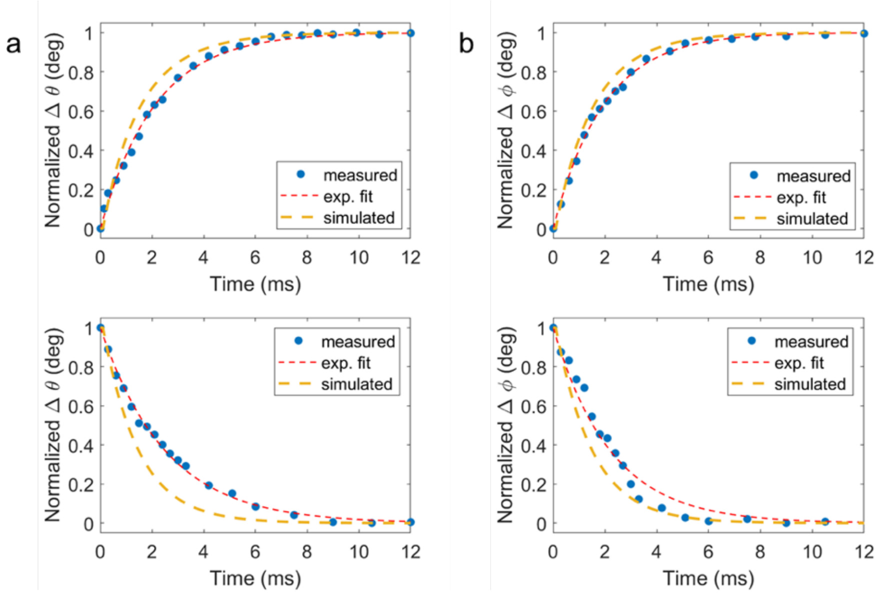

Fig. S5: Time response of the L-shaped cantilever. Measured and simulated rise time (top) and fall time (bottom) of the secondary arm (**a**) and primary arm (**b**) of the L-shaped cantilever.

5. Improving the resolution by modifying the grating coupler design

The number of resolvable points of our devices was limited due to the short length (25 µm) of the grating couplers, which led to a relatively large divergence angle (FWHM = 1.4°). The number of resolvable points, at least in the longitudinal direction, could be significantly increased without modification of the cantilever design by increasing the grating coupler length along the cantilever and reducing the scattering strength of the grating. To quantitatively illustrate the effectiveness of this approach, based on the analytical model explained in Chapter 6 of [10], we calculated the FWHM of the output beam at λ=488 nm as a function of grating coupler length (L_{GC}) and the scattering strength (α) of the gratings. The calculation results of the analytical model are shown in Fig. S6a for L_{GC} between 5 µm and 2 mm. For large values of the scattering strength (e.g., blue curve in Fig. S6a), increasing the grating length beyond the point where the light is fully coupled out does not decrease the FWHM of the output beam. This is the case for single layer fully-etched grating couplers, which were used in this work. However, by using sidewall gratings, it is possible to effectively increase the grating length and reduce the FWHM. To validate the analytical method, we simulated a 100 µm long and 4 µm wide SiN grating coupler with additional 2 µm wide sidewall gratings, using the same thickness of 150 nm, using 3D FDTD simulation in Lumerical. The resulting output beam in the propagation direction is shown in Fig. S6b corresponding to the green dot in Fig. S6a. Compared to the grating couplers we used (red dot in Fig. 6Sa) the simulated grating coupler has a 4-fold improvement in the FWHM in the longitudinal dimension. The design flexibility of the demonstrated cantilevers allows for tuning the properties of the output beam for a certain application. For instance, since a high angular

resolution is desirable for head-mounted augmented reality displays, the length of the grating can be increased to 1 mm (see Fig. S6a) to each a beam size of 0.02°.

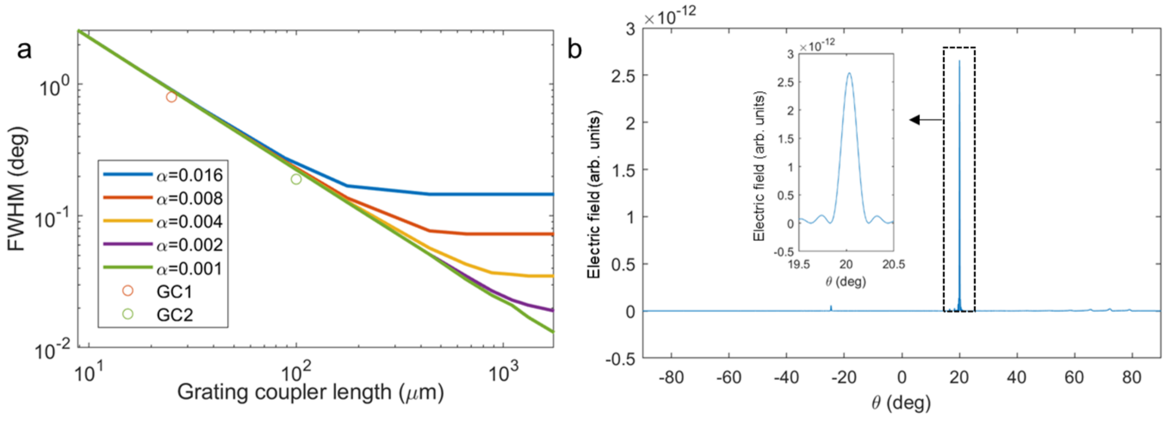

Fig. S6: Simulated performance of a weaker grating coupler. a Calculated full-width-half-maximum of the output beam of the grating coupler according to its length and scattering strength. The red and green dots, respectively, represent the measured 25 μm long grating coupler (GC1) and the simulated and the simulated 100 μm long grating coupler (GC1) **b** Simulated far-field profile of the output beam at λ=488 nm for a 100 μm long grating coupler with α=0.002.

Long cantilevers nominally have longer rise/fall times. One way to overcome the lower speed of the long cantilevers is to overdrive the device. This method has been previously used to get a faster time-response in thermal phase tuners [11]. Fig. S7(b) shows the simulated fall time of a 1-mm long rectilinear cantilever, under the applied voltage in Fig. S7(a). The fall time is reduced to 0.5 ms, about an order of magnitude lower than the nominal fall time with a simple step function drive power. To improve the rise time, without a thermoelectric cooler, large metal patches should be used on the clamped side of the cantilever to better heat sinking.

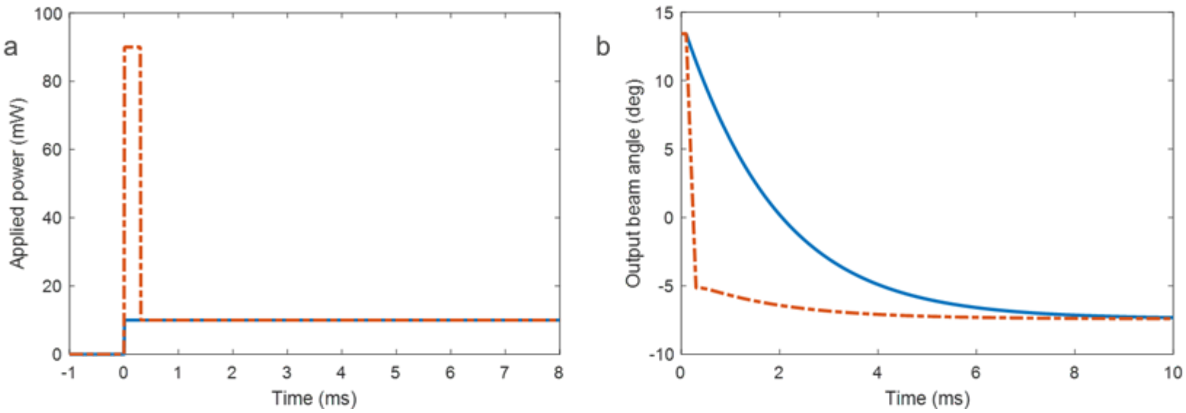

Fig. S7: Simulated fall time with overdriving. a Applied electrical power. The dashed red curve shows the applied power with an overdrive, which is 90 mW before t = 0.3 ms. The blue curve shows a simple step function. **b** Simulated output beam angle as a result of two types of input power signals. Overdriving the applied power shortens the fall time to 0.5 ms.

6. Cryogenic measurement setup of the L-shaped microcantilever

The characterization setup used for cryogenic measurements is shown in Fig. S8a. The cryostat had an open window of 10 cm in diameter at the top that allowed us to view and measure the emitted beam. The optical and electrical packaging of the PIC were performed prior to the measurements as explained in the Methods section of the main manuscript. Generally, electro-thermally tuned optical devices are not designed to perform in cryogenic conditions. For instance, the high thermal budget of the state-of-the-art SiN beam formers [12] can easily overwhelm the cooling power of the cryostat which is typically in the range of hundreds of milliwatts. However, due to the relatively small power consumption of our devices, the temperature could be reduced to 4K for an applied power of < 30 mW. We performed thermal simulations using COMSOL Multiphysics to calculate the temperature profile in the PIC under 26 mW of applied electrical power to the secondary arm of the L-shaped cantilever, while the chip is fixed on the cold head with T = 10 K. The results (Figs. S8b and S8c) show that at a distance of only ~ 600 µm from the cantilever, the temperature falls to 11 K, which is one degree above the set point of the cryostat. This suggests that our devices can be implemented on the same chip in conjunction with other electro-optical devices (such as single-photon detectors) which require a low-temperature condition for their performance, without any significant crosstalk, provided that an adequate clearance is maintained.

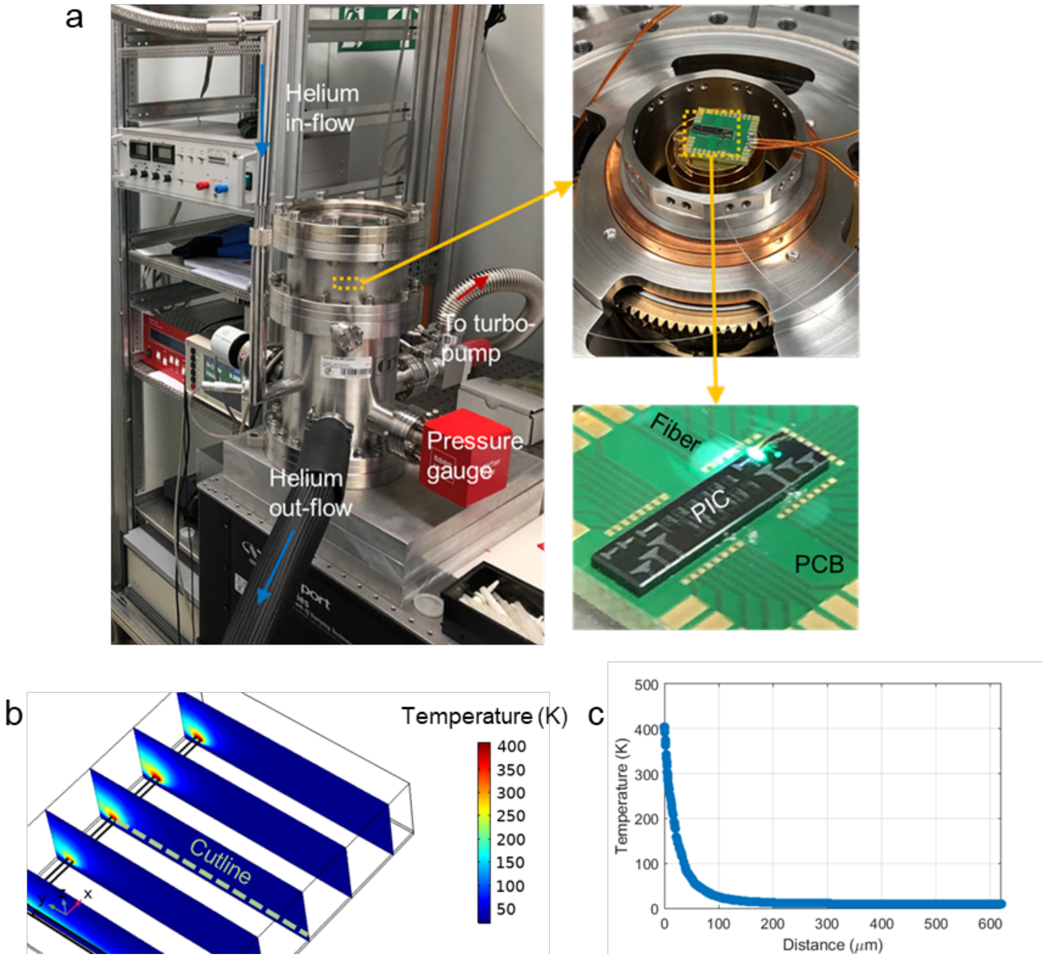

Fig. S8: a Cryogenic measurement setup**. b** Simulated temperature profile in the vicinity of the L-shaped beam scanner under 26 mW of applied electrical power. **c** Temperature along the green dashed line in **b**) showing the decay of the temperature.

7. Comparison with state-of-the-art beam steering systems

The results of the rectilinear and L-shaped cantilevers in terms of steering range, power consumption, number of resolvable spots, temporal response, and resonant frequencies are summarized in Table S1.

Table S1 | Summary of the demonstrated devices

Device	Dimension	Steering range (°)	Resolvable points	Required power (mW)	t_{rise} (ms)<br>t_{fall} (ms)	f_{res} (kHz)
L-shaped<br>(500 × 600 μm)	2D	24 × 12	66	45	4.3, 4.7<br>4.9, 5.6	7.6 (f_1)<br>17.4 (f_2)
Rectilinear<br>(300 μm)	1D	11	8	30	1.0<br>1.4	77.4
Rectilinear<br>(500 μm)	1D	17.6	12	30	2.4<br>2.8	24.8
Rectilinear<br>(800 μm)	1D	22.6	16	30	4.1<br>4.0	11.8
Rectilinear<br>(1 mm)	1D	30.1	21	30	3.6<br>5.7	5.7

Table S2 presents a comparison of this work with other beam-steering PIC demonstrations in the visible spectral range. So far, only a few PIC beam scanners have been reported. The microcantilevers presented here achieve fast beam steering without a wavelength sweep and at a record low power consumption.

Table S2 | Comparison of beam steerers in the visible spectrum

Device	Dim.	λ(nm)	Beam steering method	Steering range (°)	Resolvable points	Required power (mW)	Time response	f_{res} (kHz)	Emitter loss	Ref.
L-shaped cantilever	2D	410 - 700[1]	θ: MEMS<br>φ: MEMS	24 × 12	66	θ: 23<br>φ: 23	~5 ms	7.6 (f_1)<br>17.4 (f_2)	5.2 dB	This work
Optical phased array	1D	520 - 980	θ: Wavelength sweep	65	~260[2]	Not reported	Not reported	NA	Not reported	[13]
Optical phased array	1D	488	φ: Thermal tuners	50	~294[2]	φ: 2000	Not reported	NA	Not reported	[12]
Optical phased array	2D	650 - 980	θ: Wavelength sweep<br>φ: Thermal tuners	44 × 13	Not reported[3]	θ: Not rep.<br>φ: Not rep.	Not reported	NA	Not reported	[14]

[1] Two-dimensional beam steering can be achieved at any target wavelength in this range.
[2] Calculated based on the reported FWHM.
[3] FWHM in the longitudinal direction is reported to be 3.4° at λ = 850 nm.

Supplementary References

1. S. Timoshenko, "Analysis of bi-metal thermostats," J. Opt. Soc. Am. **11**, 233–255 (1925).
2. Y. Zhang, A. Toda, H. Okada, T. Kobayashi, T. Itoh, and R. Maeda, "New wafer-scale MEMS fabrication of 3D silicon/metal cantilever array sensor," in *2012 IEEE 25th International Conference on Micro Electro Mechanical Systems (MEMS)* (2012), pp. 297–300.
3. R. Prajesh, B. Shankar, N. Jain, A. Agarwal, R. Prajesh, and A. Agarwal, "A quick method to realize and characterize bimorph cantilevers," in *2014 IEEE 2nd International Conference on Emerging Electronics (ICEE)* (2014), pp. 1–3.
4. P. Wang, Y. Liu, D. Wang, H. Liu, W. Liu, and H. Xie, "Stability study of an electrothermally-actuated MEMS mirror with Al/SiO2 bimorphs," Micromachines **10**, (2019).
5. W. Liao, E. X. Zhang, M. L. Alles, A. L. Sternberg, C. N. Arutt, D. Wang, S. E. Zhao, P. Wang, M. W. McCurdy, H. Xie, D. M. Fleetwood, R. A. Reed, and R. D. Schrimpf, "Total-ionizing-dose effects on Al/SiO2 bimorph electrothermal microscanners," IEEE Trans. Nucl. Sci. **65**, 2260–2267 (2018).
6. K. Jia, S. Pal, and H. Xie, "An electrothermal tip–tilt–piston micromirror based on folded dual S-shaped bimorphs," J. Microelectromech. Syst. **18**, 1004–1015 (2009).
7. M. Jacques, A. Samani, E. El-Fiky, D. Patel, Z. Xing, and D. V. Plant, "Optimization of thermo-optic phase-shifter design and mitigation of thermal crosstalk on the SOI platform," Opt. Express **27**, 10456–10471 (2019).
8. L. Jia, C. Li, T.-Y. Liow, and G.-Q. Lo, "Efficient suspended coupler with loss less than -1.4 dB

between Si-photonic waveguide and cleaved single mode fiber," J. Lightwave Technol. **36**, 239–244 (2018).
9. W. Peng, Z. Xiao, and K. R. Farmer, "Optimization of thermally actuated bimorph cantilevers for maximum deflection," Nanotech Proceedings **1**, 376–379 (2003).
10. C. A. Balanis, *Antenna Theory: Analysis and Design* (John Wiley & Sons, 2005).
11. M. W. Geis, S. J. Spector, R. C. Williamson, and T. M. Lyszczarz, "Submicrosecond submilliwatt silicon-on-insulator thermooptic switch," IEEE Photonics Technol. Lett. **16**, 2514–2516 (2004).
12. M. C. Shin, A. Mohanty, K. Watson, G. R. Bhatt, C. T. Phare, S. A. Miller, M. Zadka, B. S. Lee, X. Ji, I. Datta, and M. Lipson, "Chip-scale blue light phased array," Opt. Lett. **45**, 1934–1937 (2020).
13. H. Wang, Z. Chen, C. Sun, S. Deng, X. Tang, L. Zhang, R. Jiang, W. Shi, Z. Chen, Z. Li, and A. Zhang, "Broadband silicon nitride nanophotonic phased arrays for wide-angle beam steering," Opt. Lett. **46**, 286–289 (2021).
14. C. Sun, L. Yang, B. Li, W. Shi, H. Wang, Z. Chen, X. Nie, S. Deng, N. Ding, and A. Zhang, "Parallel emitted silicon nitride nanophotonic phased arrays for two-dimensional beam steering," Opt. Lett. **46**, 5699–5702 (2021).
